# Segmenting Brain Tumor Using Cascaded V-Nets in Multimodal MR Images

**DOI:** 10.3389/fncom.2020.00009

**Published:** 2020-02-14

**Authors:** Rui Hua, Quan Huo, Yaozong Gao, He Sui, Bing Zhang, Yu Sun, Zhanhao Mo, Feng Shi

**Affiliations:** ^1^School of Biological Science and Medical Engineering, Southeast University, Nanjing, China; ^2^Shanghai United Imaging Intelligence, Co., Ltd., Shanghai, China; ^3^China-Japan Union Hospital of Jilin University, Changchun, China; ^4^Department of Radiology, Affiliated Drum Tower Hospital of Nanjing University Medical School, Nanjing, China

**Keywords:** deep learning, brain tumor, segmentation, V-Net, multimodal, magnetic resonance imaging

## Abstract

In this work, we propose a novel cascaded V-Nets method to segment brain tumor substructures in multimodal brain magnetic resonance imaging. Although V-Net has been successfully used in many segmentation tasks, we demonstrate that its performance could be further enhanced by using a cascaded structure and ensemble strategy. Briefly, our baseline V-Net consists of four levels with encoding and decoding paths and intra- and inter-path skip connections. Focal loss is chosen to improve performance on hard samples as well as balance the positive and negative samples. We further propose three preprocessing pipelines for multimodal magnetic resonance images to train different models. By ensembling the segmentation probability maps obtained from these models, segmentation result is further improved. In other hand, we propose to segment the whole tumor first, and then divide it into tumor necrosis, edema, and enhancing tumor. Experimental results on BraTS 2018 online validation set achieve average Dice scores of 0.9048, 0.8364, and 0.7748 for whole tumor, tumor core and enhancing tumor, respectively. The corresponding values for BraTS 2018 online testing set are 0.8761, 0.7953, and 0.7364, respectively. We also evaluate the proposed method in two additional data sets from local hospitals comprising of 28 and 28 subjects, and the best results are 0.8635, 0.8036, and 0.7217, respectively. We further make a prediction of patient overall survival by ensembling multiple classifiers for long, mid and short groups, and achieve accuracy of 0.519, mean square error of 367240 and Spearman correlation coefficient of 0.168 for BraTS 2018 online testing set.

## Introduction

Gliomas are the most common brain tumors and comprise about 30 percent of all brain tumors. Gliomas occur in the glial cells of the brain or the spine (Mamelak and Jacoby, 2007). They can be further categorized into low-grade gliomas (LGG) and high-grade gliomas (HGG) according to their pathologic evaluation. LGG are well-differentiated and tend to exhibit benign tendencies and portend a better prognosis for the patients. HGG are undifferentiated and tend to exhibit malignant and usually lead to a worse prognosis. With the development of the magnetic resonance imaging (MRI), multimodal MRI plays an important role in disease diagnosis. Different MRI modalities are sensitive to different tumor tissues. For example, T2-weighted (T2) and T2 Fluid Attenuation Inversion Recovery (FLAIR) are sensitive to peritumoral edema, and post-contrast T1-weighted (T1Gd) is sensitive to necrotic core and enhancing tumor core. Thus, they can provide complementary information about gliomas.

Segmentation of brain tumor is a prerequisite while essential task in disease diagnosis, surgical planning and prognosis (Bakas et al., 2017a). Automatic segmentation provides quantitative information that is more accurate and has better reproducibility than conventional qualitative image review. Moreover, the following task of brain tumor classification heavily relies on the results of brain tumor segmentation. Automatic segmentation is considered as a powered engine and empower other intelligent medical application. However, the segmentation of brain tumor in multimodal MRI scans is one of the most challenging tasks in medical imaging analysis due to their highly heterogeneous appearance, and variable localization, shape and size.

Before deep learning developed, random forest (RF) achieves better performance in brain tumor segmentation (Zikic et al., 2012; Le Folgoc et al., 2016). In recent years, with the rapid development of deep leaning techniques, state-of-the-art performance on brain tumor segmentation have been achieved with convolutional neural network (CNN). For example, in Cui et al. (2018), an end-to-end training using fully convolutional network (FCN) showed satisfactory performance in the localization of the tumor, and patch-wise CNN was used to segment the intra-tumor structure. In Wang et al. (2018), a cascaded anisotropic CNN was designed to segment three sub-regions with three Nets, and the segmentation result from previous net was used as receptive field in the next net. Ensemble strategy also shows great advantages, and most models are based on 3D U-Net, DeepMedic, and their variants (Isensee et al., 2018; Kamnitsas et al., 2018). One recent paper arguing that a well-trained U-Net is hard to beat (Isensee et al., 2019). Instead of modifying architectures, they focused on the training process such as region based training and additional training data, and achieved competitive Dice scores.

Inspired by the superior performance of V-Net in segmentation tasks, we propose a cascaded V-Nets method to segment brain tumor into three substructures and background. In particular, the cascaded V-Nets not only take advantage of residual connection but also use the extra coarse localization and ensemble of multiple models to boost the performance. A preliminary version of the method has been presented in a conference (Hua et al., 2019). Here we extend it to include more descriptions of the method details and additional experiments to further evaluate the performance of the proposed method in local hospital data sets.

## Method

### Dataset and Preprocessing

The data used in experiments come from the released data of BraTS 2018 online challenge (Menze et al., 2015; Bakas et al., 2017a,b,c). The training set includes totally 210 HGG patients and 75 LGG patients. The validation set includes 66 patients and the testing set includes 191 patients. Each patient has four MRI modalities including T1-weighted (T1), T2, T1Gd, and FLAIR, where ground truth labels of tumor substructures are available only in training set. The images were already skull stripped and normalized together, with resolution of 1 × 1 × 1 mm^3^ for all modalities. We use 80 percent of the training data for our training, and the rest 20 percent of the training data as our local testing set.

Meanwhile, in order to further test the performance of the proposed method, we prepare two additional data sets that include 28 patients from China-Japan Union Hospital of Jilin University and another 28 patients from Affiliated Drum Tower Hospital of Nanjing University Medical School. The resolution of the T1 images from China-Japan Union Hospital of Jilin University is 0.6 × 0.6 × 6 mm^3^, while the resolution of the T1 images from Affiliated Drum Tower Hospital of Nanjing University Medical School is 0.67 × 0.67 × 0.67 mm^3^. The images of T2, T1Gd, and FLAIR are linearly aligned to its corresponding T1 image for each subject. Skull stripping is performed on T1 and the mask is applied to other modalities. The ground truth labels of the brain tumors are manually delineated by an experienced radiologist. The experienced radiologist (Z.M.) was asked to delineate the tumor subregions according to the image delineating principles of BraTS 2018. Results would serve as ground truth to evaluate the generalizability of the method. In detail, the delineating principle includes three subregion segmentations of the tumor, including the necrotic (NCR) and the non-enhancing (NET) tumor core, the enhancing tumor (ET) and the peritumoral edema (ED). The NCR and the NET tumor core was the low intensity necrotic structures in T1Gd when compared to T1. The ET area was confirmed as hyper-intensity structures in T1Gd when compared to T1 images, and when compared to normal brain in T1Gd. The ED area was identified as abnormality visible in T2 and FLAIR excluding ventricles and cerebrospinal fluid.

**Figure 1:**
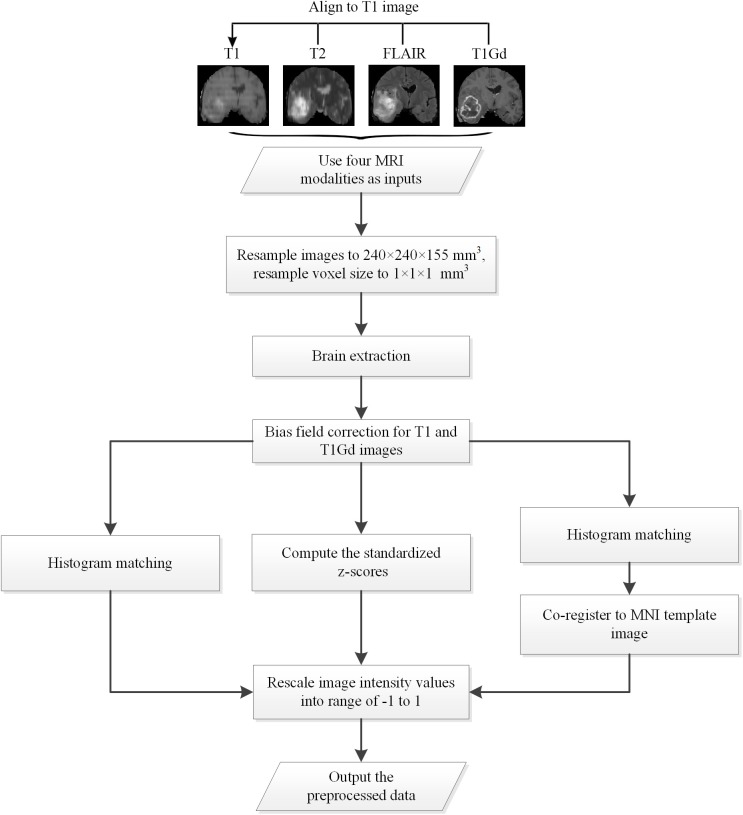
The flow chart of the preprocessing procedures.

All data used in the experiments are preprocessed with specific designed procedures. A flow chart of the proposed preprocessing procedures is shown in Figure 1, as follows: (1) Apply bias field correction N4 (Tustison et al., 2010) to T1 and T1Gd images, normalize each modality using histogram matching with respect to a MNI template image, and rescale the images intensity values into range of −1 to 1; (2) Apply bias field correction N4 to all modalities, compute the standardized z-scores for each image and rescale 0–99.9 percentile intensity values into range of −1 to 1; (3) Follow the first method, and further apply affine alignment to co-register each image to the MNI template image.

### V-Net Architecture

**Figure 2:**
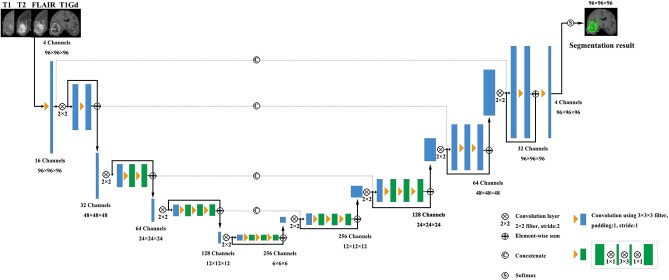
The architecture of the used V-Net.

V-Net was initially proposed to segment prostate by training an end-to-end CNN on MRI (Milletari et al., 2016). The architecture of our V-Net is shown in Figure 2. The left side of V-Net reduces the size of the input by down-sampling, while the right side of V-Net recovers the semantic segmentation image that has the same size with input images by applying de-convolutions. The detailed parameters about V-Net is shown in Table 1. Both left side of the network and right side of the network were divided into four blocks that operate at different resolutions. Each block comprises one to three convolutional blocks. The input of each block is added to the output of the current block to learn a residual function, and added to the input of the corresponding block which has the same resolution in the right side of the network as a skip connection. By means of introducing residual function and skip connection, V-Net has better segmentation performance compared with conventional CNN. Each convolutional block comprises two convolutional layers with the kernel size of 1 × 1 × 1 at the start and the end of the convolutional block. By means of introducing the 3D kernel with size of 1 × 1 × 1, the number of parameters in V-Net is decreased and the memory consumption is greatly reduced. Appropriate padding and ReLU non-linearity are applied throughout the network.

**Table 1:** The detailed parameters of the used V-Net, as shown in Figure 2.

**Blocks**	**Sub-blocks or layers**	**Input dimensions**	**Output dimensions**
Input block	Conv(k = 3, *p* = 1, s = 1) + BN + ReLU	96 × 96 × 96 × 4	96 × 96 × 96 × 16
Down block 1	Conv(k = 2, *p* = 0, s = 2)+ BN + ReLU	96 × 96 × 96 × 16	48 × 48 × 48 × 32
	Conv(k = 3, *p* = 1, s = 1) + BN^*^	48 × 48 × 48 × 32	–
	(input+output) + ReLU^*^	48 × 48 × 48 × 32	–
Down block 2	Conv(k = 2, *p* = 0, s = 2) + BN + ReLU	48 × 48 × 48 × 32	24 × 24 × 24 × 64
	Conv block × 2^*^	24 × 24 × 24 × 64	–
	(input+output) + ReLU^*^	24 × 24 × 24 × 64	–
Down block 3	Conv(k = 2, *p* = 0, s = 2) + BN + ReLU	24 × 24 × 24 × 64	12 × 12 × 12 × 128
	Conv block × 3^*^	12 × 12 × 12 × 128	–
	(input+output) + ReLU^*^	12 × 12 × 12 × 128	–
Down block4	Conv(k = 2, *p* = 0, s = 2) + BN + ReLU	12 × 12 × 12 × 128	6 × 6 × 6 × 256
	Conv block × 3^*^	6 × 6 × 6 × 256	–
	(input+output) + ReLU^*^	6 × 6 × 6 × 256	–
Up block 1	Conv(k = 2, *p* = 0, s = 2) + BN + ReLU	6 × 6 × 6 × 256	12 × 12 × 12 × 128
	Cat(output, skip)^*^	12 × 12 × 12 × 128	12 × 12 × 12 × 256
	Conv block × 3^*^	12 × 12 × 12 × 256	–
	(input+output) + ReLU^*^	12 × 12 × 12 × 256	–
Up block 2	Conv(k = 2, *p* = 0, s = 2) + BN + ReLU	12 × 12 × 12 × 256	24 × 24 × 24 × 64
	Cat(output+skip)^*^	24 × 24 × 24 × 64	24 × 24 × 24 × 128
	Conv Block × 3^*^	24 × 24 × 24 × 128	–
	(input+output) + ReLU^*^	24 × 24 × 24 × 128	–
Up block 3	Conv(k = 2, *p* = 0, s = 2) + BN + ReLU	24 × 24 × 24 × 128	48 × 48 × 48 × 32
	Cat(output+skip)^*^	48 × 48 × 48 × 32	48 × 48 × 48 × 64
	Conv(k = 3, *p* = 1, s = 1) + BN + ReLU^*^	48 × 48 × 48 × 64	–
	Conv(k = 3, *p* = 1, s = 1)+BN^*^	48 × 48 × 48 × 64	–
	(input+output) + ReLU^*^	48 × 48 × 48 × 64	–
Up block 4	Conv(k = 2, *p* = 0, s = 2) + BN + ReLU	48 × 48 × 48 × 64	96 × 96 × 96 × 16
	Cat(output+skip)^*^	96 × 96 × 96 × 16	96 × 96 × 96 × 32
	Conv(k = 3, *p* = 1, s = 1) + BN^*^	96 × 96 × 96 × 32	–
	(input+output) + ReLU^*^	96 × 96 × 96 × 32	–
Out block	Conv(k = 1, *p* = 0, s = 1) + BN + ReLU	96 × 96 × 96 × 32	96 × 96 × 96 × 4
	Softmax	96 × 96 × 96 × 4	96 × 96 × 96 × 1

Each Conv sub-block contains three convolution layers: Conv1 (k = 1, p = 0, s = 1), Conv2 (k = 3, p = 1, s = 1), and Conv3 (k = 1, p = 0, s = 1). k, kernel size; p, padding; s, stride. The symbol “–” means the output dimensions are the same with input dimensions. The symbol “^*^” denotes that these layers in each block are residual units.

### Proposed Cascaded V-Nets Framework

Although V-Net has demonstrated promising performance in segmentation tasks, it could be further improved if incorporated with extra information, such as coarse localization. Therefore, we propose a cascaded V-Nets method for tumor segmentation. Briefly, we (1) use one V-Net for the whole tumor segmentation; (2) use a second V-Net to further divide the tumor regions into three substructures, e.g., tumor necrosis, edema, and enhancing tumor. Note that the coarse segmentation of whole tumor in the first V-Net is also used as receptive field to boost the performance. Detailed steps are as follows.

**Figure 3:**
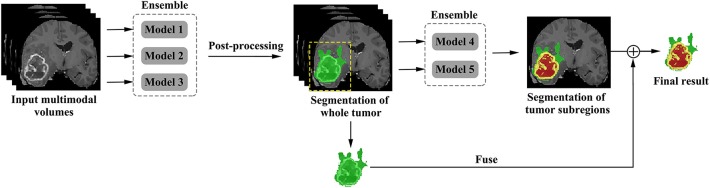
The proposed framework of cascaded V-Nets for brain tumor segmentation.

The proposed framework is shown in Figure 3. There are two networks to segment substructures of brain tumors sequentially. The first network (V-Net 1) includes models 1–3, designed to segment the whole tumor. These models are trained by three kinds of preprocessed data mentioned in part of 2.1, respectively. V-Net 1 uses four modalities MR images as inputs, and outputs the mask of whole tumor (WT). The second network (V-Net 2) includes models 4–5, designed to segment the brain tumor into three substructures: tumor necrosis, edema, and enhancing tumor. These models are trained by the first two kinds of preprocessed data mentioned in part of 2.1, respectively. V-Net 2 also uses four modalities MR images as inputs, and outputs the segmented mask with three labels. Note that the inputs of V-Net 2 have been processed using the mask of WT as region of interest (ROI). In other words, the areas out of the ROI are set as background. Finally, we combine the segmentation results of whole tumor obtained by V-Net 1 and the segmentation results of tumor core (TC, includes tumor necrosis and enhancing tumor) obtained by V-Net 2 to achieve more accurate results about the three substructures of brain tumor. In short, the cascaded V-Nets take advantage of segmenting the brain tumor and three substructures sequentially, and ensemble of multiple models to boost the performance and achieve more accurate segmentation results.

### Ensemble Strategy

We employ a simple yet efficient ensemble strategy. It works by averaging the probability maps obtained from different models. We use ensemble strategy twice in the two-step segmentation of the brain tumor substructures. For example, in V-Net 1, the probability maps of WT obtained from model 1, model 2, and model 3 were averaged to get the final probability map of WT. In V-Net 2, the probability maps of tumor necrosis, edema, and enhancing tumor obtained from model 4 and model 5 were averaged to get final probability maps of brain tumor substructures, respectively. In order to evaluate the effect of ensemble strategy for enhancing the performance of our cascaded V-Nets, ablation experiments were conducted on MICCAI BraTS 2018 validation dataset. Briefly, model combinations include Model 1–4, Model 12–4, Model 123–4, Model 123–45, and Model 123–45-fuse. To evaluate the significance of the results between different model combinations, we first evaluated the overall difference across model combinations with Kruskal-Wallis H test, and then checked the difference between each of two groups with Mann-Whitney U test. Multiple comparison correction was performed using Bonferroni criteria.

### Network Implementation

Our cascaded V-Nets are implemented in the deep learning framework PyTorch. In our network, we initialize weights with kaiming initialization (He et al., 2015), and use focal loss (Lin et al., 2018) illustrated in formula (1) as loss function. Focal loss has the advantage of balancing the ratio of positive and negative samples, and decreases the importance of easy classified samples to focus more on difficult samples (Lin et al., 2018). Adaptive Moment Estimation (Adam) (Kingma and Ba, 2014) is used as optimizer with learning rate of 0.001, and batch size of 8. Experiments are performed with a NVIDIA Titan Xp 12GB GPU.

(1) $$Focal\_Loss\left(p_{t}\right)=-\alpha {\left(1-p_{t}\right)}^{r}log\left(p_{t}\right)$$

where, α denotes the weight to balance the importance of positive/negative samples, *r* denotes the factor to increase the importance of correcting misclassified samples, and *p*_*t*_ denotes the probability of the ground truth.

In order to reduce the memory consumption in the training process, 3D patches with a size of 96 × 96 × 96 are used. And the center of the patch is confined to the bounding box of the brain tumor. Therefore, every patch used in training process contains both tumor and background. The training efficiency of the network has been greatly improved.

### Post-processing

The predicted tumor segmentations are post-processed using connected component analysis. We consider that the isolated segmentation labels with small size are prone to artifacts and thus remove them. Our strategy is as follows. After the V-Net 1, the small clusters with voxel number < T = 1,000 are directly discarded. For each cluster with size between 1,000 and 15,000, its average probability of being a tumor is calculated. This cluster will be retained if the probability is no <0.85 and removed otherwise. The rest big clusters with voxel number over T = 15,000 are also retained. A binary whole tumor map is thus obtained. After the V-Net 2, we also calculated the connected component and removed the small clusters with voxel number <1,000. While if all cluster sizes are <1,000, the largest cluster will be retained.

### Evaluation of Tumor Segmentation Performance

The models trained by MICCAI BraTS 2018 training data are applied to our local testing set, MICCAI BraTS 2018 validation set, MICCAI BraTS 2018 testing set, and the additional clinical testing sets. In order to evaluate the performance of our method, Dice score, sensitivity, and specificity are calculated for whole tumor, tumor core and enhancing tumor, respectively. Dice score indicates the ratio of the area where the segmentation image intersects with the ground truth image to the total areas. Sensitivity indicates the ratio of the detected tumor voxels to all tumor voxels. Specificity indicates the ratio of the detected background voxels to all background voxels. The evaluation results for MICCAI BraTS 2018 validation set and testing set are provided by the organizer of the BraTS 2018 online challenge, and Hausdorff95 is also included, which indicates the distances of the two tumor voxels sets with a percentile value of 95%.

(2) $$Dice=\frac{2\left|A\cap B\right|}{\left|A\right|+\left|B\right|}$$

(3) $$Sensitivity=\frac{TP}{TP+FN}$$

(4) $$Specificity=\frac{TN}{TN+FP}$$

(5) $$\begin{array}{l} Hausdorff95=max[\underset{a\in A}{max\text{(95\%)}}\;\underset{b\in B}{min}\left\|a-b\right\|,\\ \;\underset{b\in B}{max\text{(95\%)}}\;\underset{a\in A}{min}\left\|b-a\right\|] \end{array}$$

where, *A* denotes the segmentation image, *B* denotes the ground truth image, *TP* denotes the number of the true positive voxels, *FN* denotes the number of the false negative voxels, *TN* denotes the number of the true negative voxels, and *FP* denotes the number of the false positive voxels.

For the additional testing sets of local hospitals, only Dice scores are evaluated. Given that the images from two data sets have different resolution, we calculate the average Dice scores for whole tumor, tumor core and enhancing tumor in two data sets, respectively.

### Prediction of Patient Overall Survival

Overall survival (OS) is a direct measure of clinical benefit to a patient. Generally, brain tumor patients could be classified into long-survivors (e.g., >15 months), mid-survivors (e.g., between 10 and 15 months), and short-survivors (e.g., <10 months). For the multimodal MRI data, we propose to use our tumor segmentation masks and generate imaging markers through Radiomics method to predict the patient OS groups.

From the training data, we extract 40 hand-crafted features and 945 radiomics features (Isensee et al., 2018) in total. The detailed extracted features are shown in Table 2. All features are normalized into range of 0–1. Pearson correlation coefficient is used for feature selection. All features are ranked by Pearson correlation coefficient from large to small, and the top 10% features are used as the inputs of the following classifiers. We use support vector machine (SVM), multilayer perceptrons (MLP), XGBoost, decision tree classifier, linear discriminant analysis (LDA), and random forest (RF) as our classifiers in an ensemble strategy. F1-score is used as the evaluation standard. The final result is determined by the vote on all classification results. In order to reduce the bias, a 10-fold cross-validation is used. For the validation and testing data, these selected features are extracted and the prediction is made using the above models.

**Table 2:** Selected features in the training data for the prediction of patient overall survival.

**Features**	**Number of features**
Age	1
Volume of whole brain	1
Volume of whole tumor	1
Volumes of three tumor substructures	3
Ratio of the whole tumor in whole brain	1
Ratios of three tumor substructures in whole tumor	3
Extent of lesion in x, y, z directions	3
Center coordinates of the whole tumor	3
Means and variances of three tumor substructures in four MR modalities	24
First order statistics features of three tumor substructures	411
Shape-based features of three tumor substructures	78
Gray level cooccurence matrix features of three tumor substructures	180
Gray level run length matrix features of three tumor substructures	96
Neigbouring gray tone difference matrix features of three tumor substructures	96
Gray level dependence matrix features of three tumor substructures	84

## Results

### Segmentation Results on Local Testing Set of 57 Subjects

**Figure 4:**
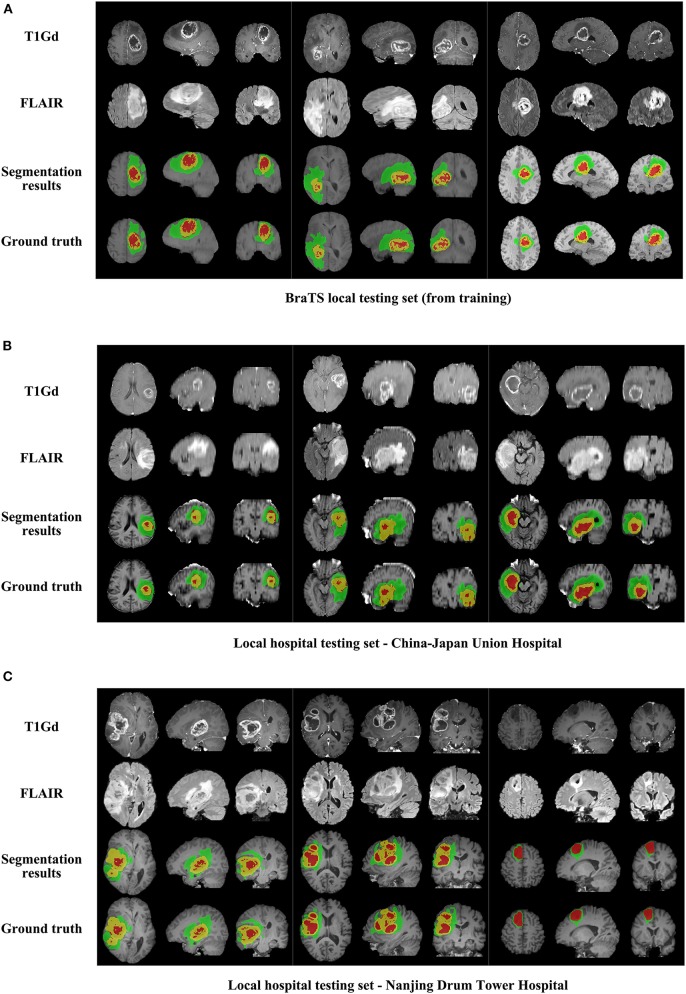
The comparison of segmentation results and ground truth on representative cases from local testing set and two clinical testing sets. **(A)** The segmentation results and ground truth from local testing set. **(B)** The segmentation results and ground truth from clinical testing set of China-Japan Union Hospital of Jilin University. **(C)** The segmentation results and ground truth from clinical testing set of Affiliated Drum Tower Hospital of Nanjing University Medical School.

We use 20 percent of all data as our local testing set, which includes 42 HGG patients and 15 LGG patients. Representative segmentation results are shown in Figure 4A. The green shows the edema, the red shows the tumor necrosis, and the yellow shows the enhancing tumor. In order to evaluate the preliminary experimental results, we calculate the average Dice scores, sensitivity, and specificity for whole tumor, tumor core, and enhancing tumor, respectively. The results are shown in Table 3. The segmentation of whole tumor achieves best result with average Dice score of 0.8505.

**Table 3:** Dice, sensitivity, and specificity measurements of the proposed method on local testing set.

	**Whole tumor**	**Tumor core**	**Enhancing tumor**
Dice mean ± SD	0.8505 ± 0.0972	0.7842 ± 0.1919	0.7426 ± 0.2080
Sensitivity mean ± SD	0.9180 ± 0.1091	0.7596 ± 0.2199	0.7174 ± 0.2337
Specificity mean ± SD	0.9981 ± 0.0012	0.9996 ± 0.0008	0.9997 ± 0.0003

### Segmentation Results on MICCAI BraTS 2018 Validation Set of 66 Subjects

**Figure 5:**
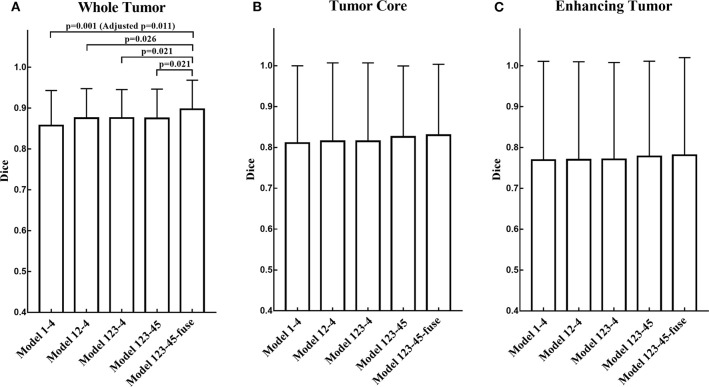
The distribution of Dice scores for whole tumor, tumor core and enhancing tumor in ablation experiments. **(A)** The bar plot of Dice scores for whole tumor. The difference between the baseline V-Nets architecture and our proposed architecture reaches significance as *p* = 0.011. **(B)** The bar plot of Dice scores for tumor core. **(C)** The bar plot of Dice scores for enhancing tumor (The height of the bar indicates the mean Dice scores, and the error bars indicate the standard deviation).

The segmentation results on BraTS 2018 online validation set achieve average Dice scores of 0.9048, 0.8364, and 0.7768 for whole tumor, tumor core, and enhancing tumor, respectively. That performance is slightly better than that in local testing set, while the whole tumor still has best result and enhancing tumor is the most challenging one. The details are shown in Table 4. For the ablation experiments, the distribution of Dice scores for whole tumor, tumor core and enhancing tumor are shown in Figures 5A–C, respectively. Generally, the average Dice scores for whole tumor, tumor core and enhancing tumor increase when ensembling more models to our cascaded V-Nets architecture. The difference of Dice scores for whole tumor between the baseline V-Nets architecture and our proposed architecture reaches significance as *p* = 0.011. Other model combination methods show the same trend although not get through Bonferroni correction.

**Table 4:** Dice, sensitivity, specificity, and Hausdorff95 measurements of the proposed method on BraTS 2018 validation set.

	**Whole tumor**	**Tumor core**	**Enhancing tumor**
Dice mean ± SD	0.9048 ± 0.0648	0.8364 ± 0.1609	0.7768 ± 0.2355
Sensitivity mean ± SD	0.9146 ± 0.0949	0.8453 ± 0.1781	0.8166 ± 0.2382
Specificity mean ± SD	0.9945 ± 0.0041	0.9971 ± 0.0041	0.9977 ± 0.0032
Hausdorff95 mean ± SD (mm)	5.1759 ± 7.3622	6.2780 ± 7.7681	3.5123 ± 4.5407

### Segmentation Results on MICCAI BraTS 2018 Testing Set of 191 Subjects

The segmentation results on BraTS 2018 online testing set achieve average Dice scores of 0.8761, 0.7953, and 0.7364 for whole tumor, tumor core and enhancing tumor, respectively. Compared with the Dice scores on MICCAI BraTS 2018 validation set, the numbers are slightly dropped. The details are shown in Table 5. The prediction of patient OS on BraTS 2018 testing set achieve accuracy of 0.519 and mean square error (MSE) of 367240. The details are shown in Table 6. The BraTS 2018 ranking of all participating teams in the testing data for both tasks has been summarized in Bakas et al. (2018), where our team listed as “LADYHR” and ranked 18 out of 61 in the segmentation task and 7 out of 26 in the prediction task.

**Table 5:** Dice and Hausdorff95 measurements of the proposed method on BraTS 2018 testing set.

	**Whole tumor**	**Tumor core**	**Enhancing tumor**
Dice mean ± SD	0.8761 ± 0.1247	0.7953 ± 0.2543	0.7364 ± 0.2592
Hausdorff95 mean ± SD (mm)	7.0514 ± 11.5935	6.7262 ± 11.8852	3.9217 ± 6.1934

**Table 6:** The prediction of patient overall survival on BraTS 2018 testing set.

	**Scores**
Accuracy	0.519
Mean squared error (MSE)	367239.974
Median square error (MedianSE)	38416
Standard deviation square error	945593.877
SpearmanR	0.168

### Segmentation Results on Clinical Testing Sets of 56 Subjects

Representative segmentation results on two local hospital testing sets are shown in Figures 4B,C. The average Dice scores for whole tumor, tumor core and enhancing tumor in two data sets are calculated, respectively. The details are shown in Table 7. Overall, the images from China-Japan Union Hospital of Jilin University which are acquired using 2D MRI sequences achieve better segmentation results with Dice scores of 0.8635, 0.8036, and 0.7217 for whole tumor, tumor core, and enhancing tumor, respectively. On the other hand, the images from Affiliated Drum Tower Hospital of Nanjing University Medical School which are acquired using 3D MRI sequences achieve poor Dice score of 0.6786 for tumor core.

**Table 7:** Dice measurements of the proposed method on clinical testing set.

	**China-Japan Union Hospital**	**Nanjing Drum Tower Hospital**
# of subjects	28	28
Image resolution (mm^3^)	0.6 × 0.6 × 6	0.67 × 0.67 × 0.67
WT Dice mean ± SD	0.8635 ± 0.0838	0.8692 ± 0.1307
TC Dice mean ± SD	0.8036 ± 0.1476	0.6786 ± 0.3093
ET Dice mean ± SD	0.7217 ± 0.1968	0.7054 ± 0.3557

## Discussion

In this paper, we propose a cascaded V-Nets framework to segment brain tumor. The cascaded framework breaks down a difficult segmentation task into two easier subtasks including segmenting whole tumor from background and segmenting tumor substructures from whole tumor. Different from other methods, our method takes full account of the effect of preprocessing on the segmentation results, and use a customized preprocessing approach to process the data and train multiple models. The cascaded V-Nets are trained only using provided data, data augmentation and a focal loss formulation. We achieve state-of-the-art results on BraTS 2018 validation set. Specifically, the experimental results on BraTS 2018 online validation set achieve average Dice scores of 0.9048, 0.8364, and 0.7768 for whole tumor, tumor core and enhancing tumor, respectively. The corresponding values for BraTS 2018 online testing set are 0.8761, 0.7953, and 0.7364, respectively.

Generally, all the three average Dice scores degenerate in testing set compared with validation set. The reason may be that the sample size of testing set is much larger than that of validation set, and includes more anatomical variances. For clinical testing sets, we achieve 2% higher average Dice scores in images acquired using 2D MRI sequences than images acquired using 3D MRI sequences. The reason may be that the public dataset provided by the organizers of MICCAI BraTS 2018 includes more images acquired using 2D MRI sequences than images acquired using 3D MRI sequences. The trained model thus favors more 2D testing data than that of 3D. However, given that 2D MRI sequences are widely adopted in clinical practice for shorter acquisition time, the generated model may be more practical and meaningful. Therefore, for sites using major 3D images, the training set could include more 3D data and a specific 3D model could be trained.

There are several benefits of using a cascaded framework. First, the cascaded framework breaks down a difficult segmentation task into two easier subtasks. Therefore, a simple network V-Net can have excellent performance. In fact, in our experiment, V-Net does have better performance when segment the tumor substructures step by step than segment background and all the three tumor substructures together. Second, the segmentation results of V-Net 1 helps to reduce the receptive field from whole brain to only whole tumor. Thus, some false positive results can be avoided.

In addition to cascaded framework, ensemble strategy contributes to the segmentation performance. In our cascaded V-Nets framework, V-Net 1 includes models 1–3 and V-Net 2 includes models 4–5. Every model uses the same network structure V-Net. However, the training data is preprocessed with different pipelines mentioned in part of 2.1. According to our experimental experience, the Dice scores will greatly decrease due to the false positive results. While we did try several ways to change the preprocessing procedures for the training data, or change the model used in the segmentation task, the false positive results always appear. Interestingly, the false positive results appear in different areas in terms of different models. Therefore, ensemble strategy works by averaging probability maps obtained from different models. The results of the ablation experiments also confirm the proposed ensemble strategy works.

Moreover, we find three interesting points in the experiment. Firstly, for multimodal MR images, the combination of data preprocessing procedures is important. In other words, different MRI modalities should be preprocessed independently. For example, in our first preprocessing pipeline, bias field correction only applied to T1 and T1Gd images. The reason is that the histogram matching approach may remove the high intensity information of tumor structure that has negative impact to the segmentation task. Secondly, we use three kinds of preprocessing methods to process the training and validation data, and compared their segmentation results. As a result, there is almost no difference between preprocessing methods in the three average Dice scores for whole tumor, tumor core and enhancing tumor, respectively. However, after the ensemble of the multiple models, the three average Dice scores all rose at least 2 percent. This suggests that data preprocessing methods is not the most important factor for the segmentation performance, while different data preprocessing methods are complementary and their combination can boost segmentation performance. Thirdly, the post-processing method is also important that it could affect the average Dices scores largely. If the threshold is too big, some of small clusters will be discarded improperly. If the threshold is too small, some false positive results will be retained. In order to have a better performance, we test a range of thresholds and choose the most suitable two thresholds as the upper and the lower bounds. For the components between upper and lower bounds, their average segmentation probabilities are calculated as a second criterion. Of course, these thresholds may not be suitable for all cases.

## Conclusions

In conclusion, we propose a cascaded V-Nets framework to segment brain tumor into three substructures of brain tumor and background. The experimental results on BraTS 2018 online validation set achieve average Dice scores of 0.9048, 0.8364, and 0.7768 for whole tumor, tumor core and enhancing tumor, respectively. The corresponding values for BraTS 2018 online testing set are 0.8761, 0.7953, and 0.7364, respectively. The corresponding values for clinical testing set are 0.8635, 0.8036, and 0.7217, respectively. For clinical data set, images acquired using 2D MRI sequences achieve higher average Dice scores than images acquired using 3D MRI sequences, demonstrates that the proposed method is practical and meaningful in clinical practice. The state-of-the-art results demonstrate that V-Net is a promising network for medical imaging segmentation tasks, and the cascaded framework and ensemble strategy are efficient for boosting the segmentation performance.

## Data Availability Statement

The datasets generated for this study are available on request to the corresponding author.

## Ethics Statement

This study was proved by the Institutional Review Board (IRB) of the Affiliated Drum Tower Hospital of Nanjing University Medical School and the China-Japan Union Hospital of Jilin University. Written informed consents were obtained from all subjects before participating to the study.

## Author Contributions

All authors listed have made a substantial, direct and intellectual contribution to the work, and approved it for publication.

### Conflict of Interest

RH, QH, YG, and FS were employed by the company of Shanghai United Imaging Intelligence, Co., Ltd., Shanghai, China. The remaining authors declare that the research was conducted in the absence of any commercial or financial relationships that could be construed as a potential conflict of interest.
